# A HOME-VISIT PILOT INTERVENTION TO PROMOTE COMMUNICATION SKILLS AND WELL-BEING FOR DEMENTIA FAMILY CAREGIVERS

**DOI:** 10.1093/geroni/igz038.2497

**Published:** 2019-11-08

**Authors:** Jung-Ah Lee, Priscilla Kehoe, Lisa Gibbs

**Affiliations:** 1 University of California Irvine, Irvine, California, United States; 2 University of California Irvine, Orange, California, United States

## Abstract

Dementia takes a significant toll on caregivers resulting in their suffering from chronic stress and depression due to responsibility for care for persons with dementia (PWD). Behaviors of PWD could be aggravated by inappropriate responses by family caregivers such as correcting PWD’s memories. The study purpose is to examine the feasibility of a home-visit-based intervention designed to promote communication skills with PWD and well-being in family caregivers. This pilot study used a single-arm experimental pre-post design to test the feasibility of 4 weekly home visits for 13 female family caregivers in Southern California (spouse, n=7; adult children, n=6; mean age=64.3, ranging 46-82). Trained home visitors used video scenarios for behavioral education for caregivers. All caregivers completed the entire home visit program. Significantly caregiver burden was decreased from baseline (M(SD)=51.38(4.58)) to follow-up at 5 weeks (M=43.31(5.67), Wilcoxon signed rank test: p=.04). Additionally, caregiver-reported PWD’s negative behaviors were reduced from baseline to follow-up (Mbase=22.31(3.52), Mfolllowup=19.31(4.4), p=.13). There were other improvements (non-significant) in greater caregiver self-efficacy and less depressive symptoms from baseline to follow-up. Caregiver satisfaction with the intervention was high (M=4.6(0.65) of 5). Qualitatively, participants appreciated the home visits for educational sessions and welcomed the empathy provided. Caregivers expressed better communications and responsiveness to the PWDs. The results showed the home-visit-based caregiver intervention was feasible and had a potential effectiveness on reduction of caregiver burden and possibly on self-efficacy and well-being. A larger-scale study will be needed to demonstrate long term positive effects on caregiver interactive skills and their well-being.

